# Cystic hypersecretory lesions - invasive breast carcinoma-spectrum of a rare tumour

**DOI:** 10.4322/acr.2023.460

**Published:** 2023-11-27

**Authors:** Saikat Mitra

**Affiliations:** 1 Unipath Speciality Laboratory Limited, Department of Histopathology and Cytopathology, Kolkata, India

**Keywords:** Breast Neoplasms, Carcinoma, Ductal, Breast, Fibrocystic Breast Disease, Hyperplasia, Secretory Component

In 1984, Rosen and Scott^1^ first described cystic hypersecretory breast lesions. This entity has a spectrum of histologically distinct lesions ranging from cystic hypersecretory hyperplasia (CHH), CHH with atypia, cystic hypersecretory carcinoma (CHC) in situ, and invasive CHC. CHH’s macroscopic examination is distinct, which shows numerous cysts of varying sizes containing gelatinous material. The cyst lining epithelium, in CHH, consists of a single layer of flattened, columnar, or cuboidal epithelium without cytological atypia. Atypical CHH shows epithelial crowding, enlarged nuclei with loss of nuclear polarity, nuclear hyperchromasia, and mitoses. The in-situ carcinoma shows intermediate to high nuclear-grade lesions with micropapillary architecture.^2^ Most reported cases are of in-situ CHC. Invasive CHC shows solid nests of poorly differentiated cells with no secretory characteristics.^3^ This rare breast cancer subtype is not included in the 5^th^ edition of the WHO breast tumor classification.

The figure above shows the macro and microscopic examination of a lesion from a 58-year-old lady who presented with a lump in her right breast for six months. Imaging revealed a large solid tumor in the upper inner quadrant of the breast. A right mastectomy was performed for this patient.

**Figure 1:**
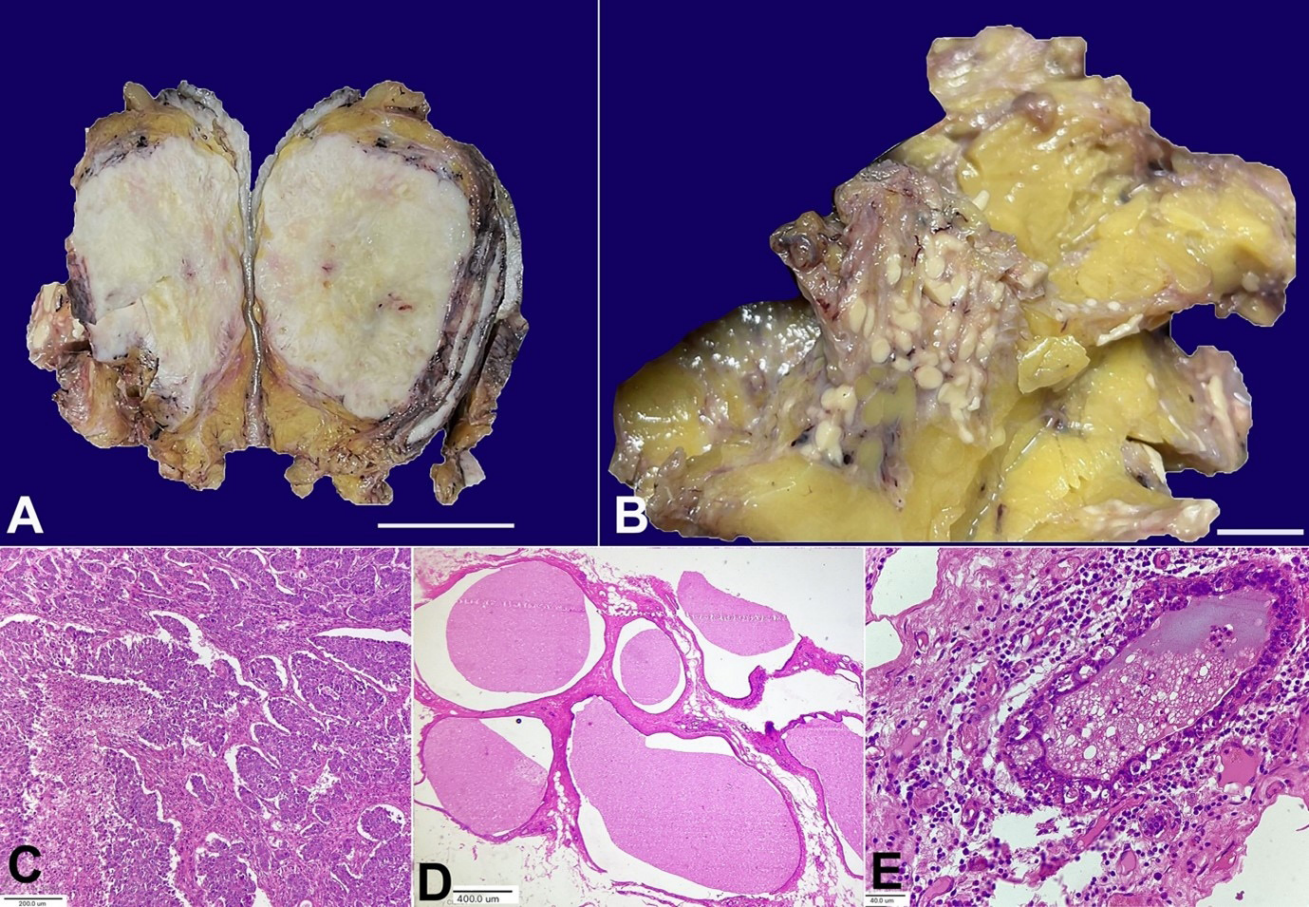
**A –** gross examination from the first lesion reveals large, well-defined solid, greyish white firm tumour of 8.5x6.5x4.0 cm (scale bar= 3 cm); **B –** gross examination from the second lesion shows multiple variable-sized yellowish-white nodules of 1.0 mm to 10.0 mm (scale bar 1 cm); **C –** photomicrograph from the solid tumour shows a cellular invasive tumour arranged in solid trabecular and sheet-like architecture with marked nuclear anaplasia and focal necrosis (H&E 100X); **D –** photomicrograph from the second lesion shows multiple cystically dilated spaces lined by cuboidal epithelium and filled with homogeneous eosinophilic colloid-like material with few macrophages, indicating CHH (H&E, 40X); **E –** photomicrograph shows CHH with nuclear atypia and epithelial multilayering (H&E 400X).

On gross inspection, the skin and nipple areola complex were unremarkable. The cut surface of the breast showed two different lesions. The first was a large, solid, well-demarcated whitish mass of 8.5x6.5x4.0 cm (Figure 1A). Additionally, an ill-defined lesion was identified in the adjacent breast parenchyma composed of clusters of multiple variable-sized yellowish nodules, ranging in size from few millimetres to 1.0 cm (Figure 1B).

The microscopic examination of the large solid mass showed a high-grade pleomorphic invasive tumor arranged in sheets and trabeculae of cells with marked nuclear pleomorphism (Figure 1C). Mitotic figures were frequent, and a focal area of necrosis was also identified. However, no microcystic or secretory changes could be identified even on examining multiple sections. Microscopic examination from the ill-defined nodular lesions showed numerous dilated cysts lined by cuboidal to columnar epithelial cells containing colloid-like eosinophilic cyst content admixed with a few foamy macrophages (Figure 1D). Occasional cysts showed nuclear atypia and epithelial multilayering (Figure 1E). However, definite in-situ carcinoma was not detected. IHC for hormone markers revealed ER and PR negative status with strong, complete membranous Her-2-neu expression in the tumor cells. Given the high-grade pleomorphic solid tumor con-existing with CHH and CHH with atypia, the invasive tumor was diagnosed as CHC.

The diagnosis of CHH can be challenging on FNA and core needle biopsy specimens. Lesions like juvenile papillomatosis, fibrocystic change, columnar cell change with or without atypia, extravasation mucocele, mucinous carcinoma, secretory carcinoma, ‘clinging’ type DCIS, and metastatic thyroid carcinoma can mimic the morphology of CHH.^4^

Although CHH is considered a benign lesion, malignant transformation on long-term follow-up has been reported.^2^ Also, there is a chance of having an in-situ or high-grade invasive tumor component with CHH. Hence, a diagnosis of CHH should alert the physician about possible association with malignancy, and a wide local excision followed by thorough sampling should be performed to look for any invasive tumor.^5^

In conclusion, cystic hypersecretory lesions rarely encountered entities with a wide spectrum of lesions, from benign to overtly malignant. Pathologists should be well versed in the gross and microscopic features and consider the possible diagnostic pitfalls while dealing with this tumor.

Although cystic hypersecretory carcinoma is not identified as a separate entity in the WHO 5th edition of breast tumor classification, associated CHH in the surrounding parenchyma indicates the invasive tumor as an invasive CHC. The available literature is limited; however, most cases of invasive CHC are high-grade solid tumors, as encountered in this case.
